# Genetic characterization and pathogenicity in a mouse model of newly isolated bat-originated mammalian orthoreovirus in South Korea

**DOI:** 10.1128/spectrum.01762-23

**Published:** 2024-01-30

**Authors:** Hai Quynh Do, Minjoo Yeom, Suyun Moon, Hanbyeul Lee, Chul-un Chung, Hee-chun Chung, Jun Won Park, Woonsung Na, Daesub Song

**Affiliations:** 1 Department of Virology, College of Veterinary Medicine and Research Institute for Veterinary Science, Seoul National University, Seoul, South Korea; 2 College of Veterinary Medicine, Chonnam National University, Gwangju, South Korea; 3 Department of Life Science, Dongguk University, Gyeongju, South Korea; 4 Department of Microbiology and Immunology, Institute for Immunology and Immunological Diseases, Yonsei University College of Medicine, Seoul, South Korea; 5 Division of Biomedical Convergence, College of Biomedical Science, Kangwon National University, Chuncheon, South Korea; Wuhan Institute of Virology, Wuhan, China

**Keywords:** *Mammalian orthoreovirus*, bat, Korea, biological characterization, pathogenicity

## Abstract

Mammalian orthoreoviruses (MRVs) infect a wide range of hosts, including humans, livestock, and wildlife. In the present study, we isolated a novel *Mammalian orthoreovirus* from the intestine of a microbat (*Myotis aurascens*) and investigated its biological and pathological characteristics. Phylogenetic analysis indicated that the new isolate was serotype 2, sharing the segments with those from different hosts. Our results showed that it can infect a wide range of cell lines from different mammalian species, including human, swine, and non-human primate cell lines. Additionally, media containing trypsin, yeast extract, and tryptose phosphate broth promoted virus propagation in primate cell lines and most human cell lines, but not in A549 and porcine cell lines. Mice infected with this strain via the intranasal route, but not via the oral route, exhibited weight loss and respiratory distress. The virus is distributed in a broad range of organs and causes lung damage. *In vitro* and *in vivo* experiments also suggested that the new virus could be a neurotropic infectious strain that can infect a neuroblastoma cell line and replicate in the brains of infected mice. Additionally, it caused a delayed immune response, as indicated by the high expression levels of cytokines and chemokines only at 14 days post-infection (dpi). These data provide an important understanding of the genetics and pathogenicity of mammalian orthoreoviruses in bats at risk of spillover infections.

**IMPORTANCE:**

Mammalian orthoreoviruses (MRVs) have a broad range of hosts and can cause serious respiratory and gastroenteritis diseases in humans and livestock. Some strains infect the central nervous system, causing severe encephalitis. In this study, we identified BatMRV2/SNU1/Korea/2021, a reassortment of MRV serotype 2, isolated from bats with broad tissue tropism, including the neurological system. In addition, it has been shown to cause respiratory syndrome in mouse models. The given data will provide more evidence of the risk of mammalian orthoreovirus transmission from wildlife to various animal species and the sources of spillover infections.

## INTRODUCTION

*Mammalian orthoreovirus* (MRV), a non-enveloped virus with a double-stranded, segmented, and linear RNA genome, belongs to the genus *Orthoreovirus* and family Reoviridae. MRVs and *Piscine orthoreovirus* are the only two species in this genus that cannot induce cell–cell fusion (1). The genome of MRVs, similar to that of other members of the genus *Orthoreovirus*, contains three large (L1–L3), three medium (M1–M3), and four small (S1–S4) segments that encode at least eight structural and three non-structural proteins (2). Based on antigenicity, MRVs are subdivided into four groups: type 1 Lang, type 2 Jones, type 3 Dearing, and type 4 Ndelle (3). MRVs are commonly detected in humans, mammals, and aquatic environments (3–8). Although MRV infections in humans have previously been considered mild or asymptomatic (9), recent studies have indicated that they may cause respiratory or gastroenteritis problems in humans, especially in children (10, 11). Additionally, mammalian orthoreoviruses have been implicated in severe encephalopathy (12–14). Similarly, mammalian orthoreovirus infections have also been reported in dogs, cats, cattle, and pigs, with clinical signs such as diarrhea and neurological problems (15–17).

Bats, a group of species with divergent characteristics such as a long life span and flying capability, have a cosmopolitan distribution, except in Antarctica (18). Furthermore, many of the species belonging to this group are gregarious and may share habitats with many different types of animals, resulting in a risk of pathogen spillover (19, 20). Bats are considered major natural reservoirs of many zoonotic viruses that cause severe diseases in humans and livestock (21–24). Serological evidence indicating a risk of spillover infection of severe acute respiratory syndrome (SARS)-related bat coronavirus in humans was observed in China (25, 26). Notably, Nipah virus and Hendra virus were detected in bat roots near the outbreak locations (21, 27). With the popularization of next-generation sequencing (NGS) technology, numerous viral clades related to significant diseases, such as SARS, Nipah, rabies, and Ebola, have been detected in various bat species (28). However, there is a gap between our knowledge of the true diversity and pathology of bat viruses. Although a large number of bat viral sequences have been published in the database, only a less number of detected viruses have been truly isolated, leading to a lack of biological information (29). Recent studies focusing on the biological and pathogenic characteristics of mammalian orthoreoviruses have revealed that bat-borne strains contain different genomic segments from different sources, such as humans, minks, and pigs (6, 30, 31). These results demonstrate that bats are important natural hosts of this virus, with potential spillover effects. Furthermore, the pathogenicity in a mouse model after artificial infection with bat-originated MRVs (32, 33) suggests the risk of novel diseases in other hosts, including animals and humans. Bat-borne orthoreoviruses are known to transmit and cause acute respiratory diseases in humans (34). Therefore, it is essential to investigate the diversity and pathology of bat orthoreoviruses to further understand their effects on human and animal health.

Virus isolation has previously been used to study viral circulation in bats in Korea. In the last decade of the 20th century, two hantavirus strains were isolated from the lung tissues of two different bat species (35). Paramyxovirus B16-40 was successfully isolated from the feces of the bat species *Miniopterus schreibersii* in 2016 (36). Recently, an MRV type 3 strain was isolated from bats; however, its biological features and pathology are not clear (37). Here, we report the isolation of a novel bat mammalian orthoreovirus from *Myotis aurascens* in Korea. This virus was isolated from the intestine of a carcass bat sample collected in Gyeongbuk province in 2020. Additionally, its biological characteristics and phylogeny were investigated. Finally, the risks of spillover and pathogenicity were examined *in vitro* and *in vivo*.

## RESULTS

### Virus isolation and characterization

**Fig 1:**
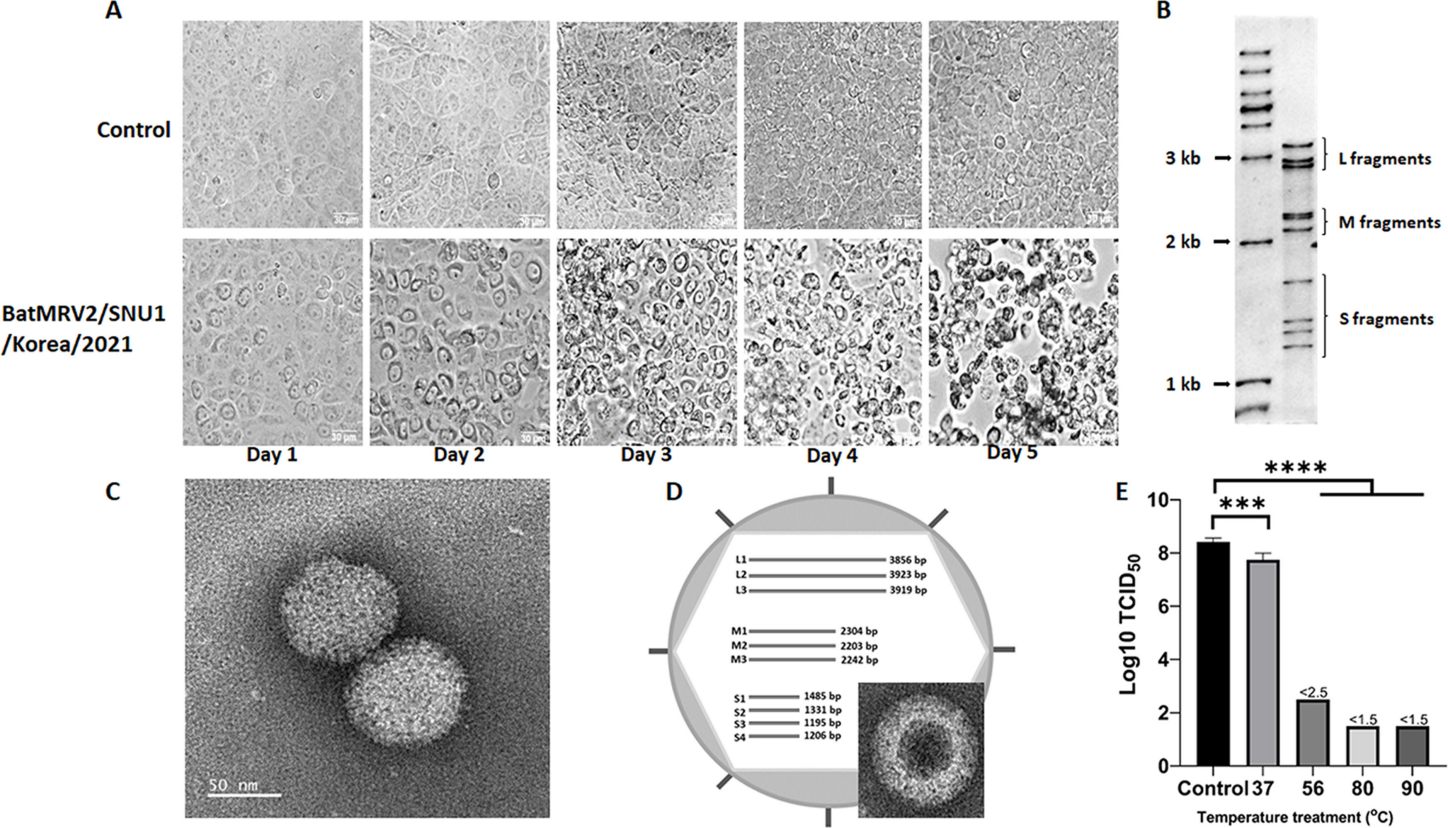
Biological characteristics of BatMRV2/SNU1/Korea/2021. (**A**) CPEs of the virus appeared in the Vero-CCL-81 cell line from day 1 to day 5 after infection (lower panel) vs mock infection (upper panel). (**B**) RNA profile of BatMRV2/SNU1/Korea/2021 on 12% gel SDS-PAGE. (**C**) Negative staining of BatMRV2/SNU1/Korea/2021 virions indicating the 80-nm-diameter particle with icosahedral morphology. (**D**) Schematic diagram of the viral genome and the double-shelled structure of the virion (inserted panel). (**E**) Virus titration after treatment in 1 h at different temperatures. The error bar indicated the standard error; statistical analysis was applied using the *t*-test, ***P < 0.001; ****P < 0.0001. The result indicated that BatMRV2/SNU1/Korea/2021 is a temperature-sensitive strain.

After two passages, the infected Vero-CCL-81 cells with small intestine sample of *M. aurascens* showed a dark ring surrounding the nucleus after 48 h (Fig. 1A). On the following day, the cells became round, detached from the surface, and clumped together (Fig. 1A). All mock-infected cells showed normal morphology. The family-specific primers for Coronaviridae, Paramyxoviridae, and Adenoviridae revealed negative results. Mycoplasma was not detected in this sample. Samples from other organs of the same or different individuals did not exhibit cytopathic effects (CPEs) after five blinded passages. A partial sequence of the S4 fragment of *Mammalian orthoreovirus* was obtained using particle-associated nucleic acid PCR (data not shown). To further confirm orthoreovirus isolation, we conducted RNA mobility analysis of double-stranded RNA segments by SDS-PAGE, which showed the ten genomic segments normally found in other *Orthoreovirus* genera (Fig. 1B). Negative-staining transmission electron microscopy of the virus collected from infected cells revealed non-enveloped icosahedral virus-like particles of approximately 70–80 nm (Fig. 1C). Additionally, a double-capsid layer structure was observed (Fig. 1D, small panel). All the aforementioned results clearly demonstrate the features of the species *Mammalian orthoreovirus* (Fig. 1D, large panel). Based on their thermal stability, mammalian orthoreoviruses are classified into heat-stable and heat-sensitive groups (38). To identify the group to which our isolate belonged, the viral solution was treated for 1 h at different temperatures. These results indicate that BatMRV2/SNU1/Korea/2021 is a heat-sensitive isolate (Fig. 1E).

### Phylogeny construction and genome characterization

In this study, by using NGS technology, the complete coding sequences (CDSs) of ten segments of BatMRV2/SNU1/Korea/2021 were obtained. Sequence comparisons indicated that L2 and M3 were highly similar to those in bat samples, whereas M2 segments were closely related to strains circulating in swine samples. Other fragments were homogenous with sequences found in strains from humans, urban environments, and wild animals, including minks and bats (Table 1).

**TABLE 1:** Highest nucleotide identities of each segment of BatMRV2/SNU1/Korea/2021 with reference strains

Genetic segment	Length (bp)	Identity (%)	MRV strain	Serotype	Host	GenBank accession number
L1	3,856	98.8	THK0325	2	Environment	LC613209.1
		98.7	17-EF40	2	Bat	MW718862.1
L2	3,923	98.4	WIV4	2	Bat	KT444533.1
		98.3	Kj22-33	2	Bat	LC752174.1
L3	3,919	96.3	WIV2	1	Bat	KT444524.1
		96.1	MRV2Tou05	2	Human	GU196308
M1	2,304	99.3	WIV5	2	Bat	KT444545.1
		97.2	THK0617	1	Environment	LC613222.1
M2	2,203	98.9	HLJYC2017	1	Swine	MN788298.1
		98.8	JS2017	1	Swine	MN788308.1
M3	2,242	98.7	WIV3	2	Bat	KT444577.1
		98.1	WIV5	2	Bat	KT444547.1
S1	1,485	98.2	THK0325	2	Environment	LC613215.1
		97.7	Osaka2005	2	Human	LC476911.1
S2	1,331	99.4	SD-14	3	Mink	KT224511.1
		99.3	WIV7	3	Bat	KT444559.1
S3	1,195	98.4	Kj22-33	2	Bat	LC752181.1
		98.4	SD-14	3	Mink	KT224512.1
S4	1,206	97.4	SD-14	3	Mink	KT224513.1
		96.5	SC-A	3	Swine	DQ396806.1

**Fig 2:**
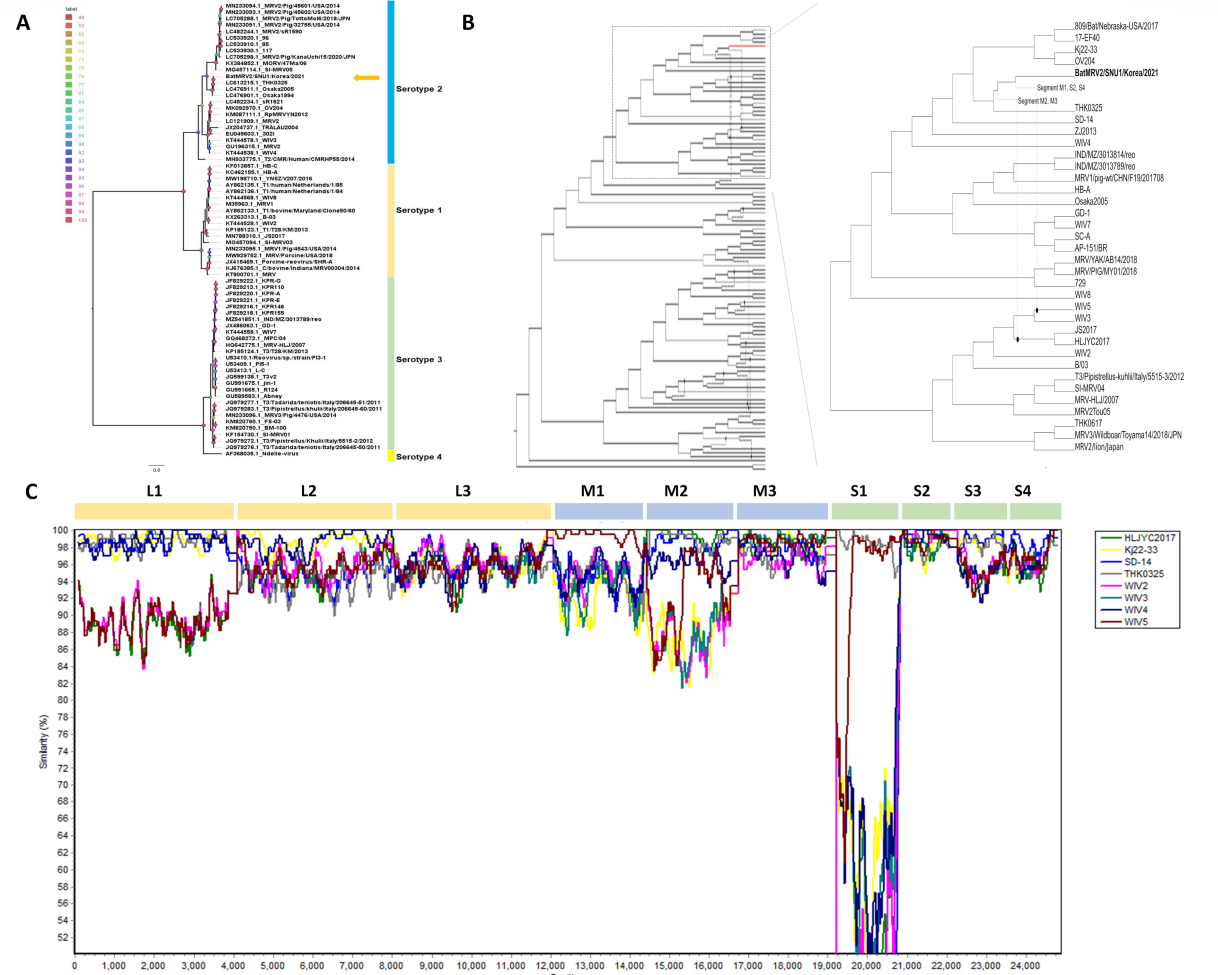
Phylogenetic construction and recombinant analysis of BatMRV2/SNU1/Korea/2021 in comparison with other published strains of MRVs. (**A**) Classification of the virus based on the Sigma 1 encoding protein revealed that this virus belonged to serotype 2. (**B**) The whole-genome phylogeny network of mammalian orthoreovirus indicated a multi-reassortment event of this isolate (indicated by the red line) with other mammalian orthoreovirus strains (left panel). The details of the reassortment event between our strain and other strains in the cluster were generated and shown in the right panel. The phylogenetic constructions of each gene are provided in Fig. S1. (**C**) Reassortment analysis of the BatMRV2/SNU1/Korea/2021 and other MRVs using Simplot.

The Sigma 1 protein is one of the major proteins contributing to the host specificity and antigenicity of *Mammalian orthoreovirus*. Phylogenetic construction based on the Sigma 1 protein-encoding sequence demonstrated that BatMRV2/SNU1/Korea/2021 belonged to serotype 2 (Fig. 2A). Interestingly, it fell within a monophyletic group containing sequences collected from strains isolated from patients and urban wastewater. Therefore, this strain may infect humans. Phylogenetic construction of the remaining segments using whole-genome sequences available in GenBank revealed that the isolated virus had close genetic relationships with other strains (Fig. S1). Additionally, the phylogenetic trees of L1, L2, M2, M3, and S3 indicated that our strain clustered with MRVs from a wide range of hosts, such as bats, humans, swine, and deer. These results suggest an intricate reassortment of MRVs.

Reassortment is a common phenomenon in segmented viruses, including *Mammalian orthoreovirus* (39). To explore the potential for gene flow, a phylogenetic network approach was applied to construct network relationships among the mammalian orthoreovirus strains. The results indicate that multiple segment exchange events occurred during the evolution of BatMRV2/SNU1/Korea/2021 (Fig. 2B). This was further supported by Simplot analysis, which revealed a difference in sequence similarity between each segment of BatMRV2/SNU1/Korea/2021 and related fragments in other reference strains.

### BatMRV2/SNU1/Korea/2021 has a broad range of cell susceptibility

**Fig 3:**
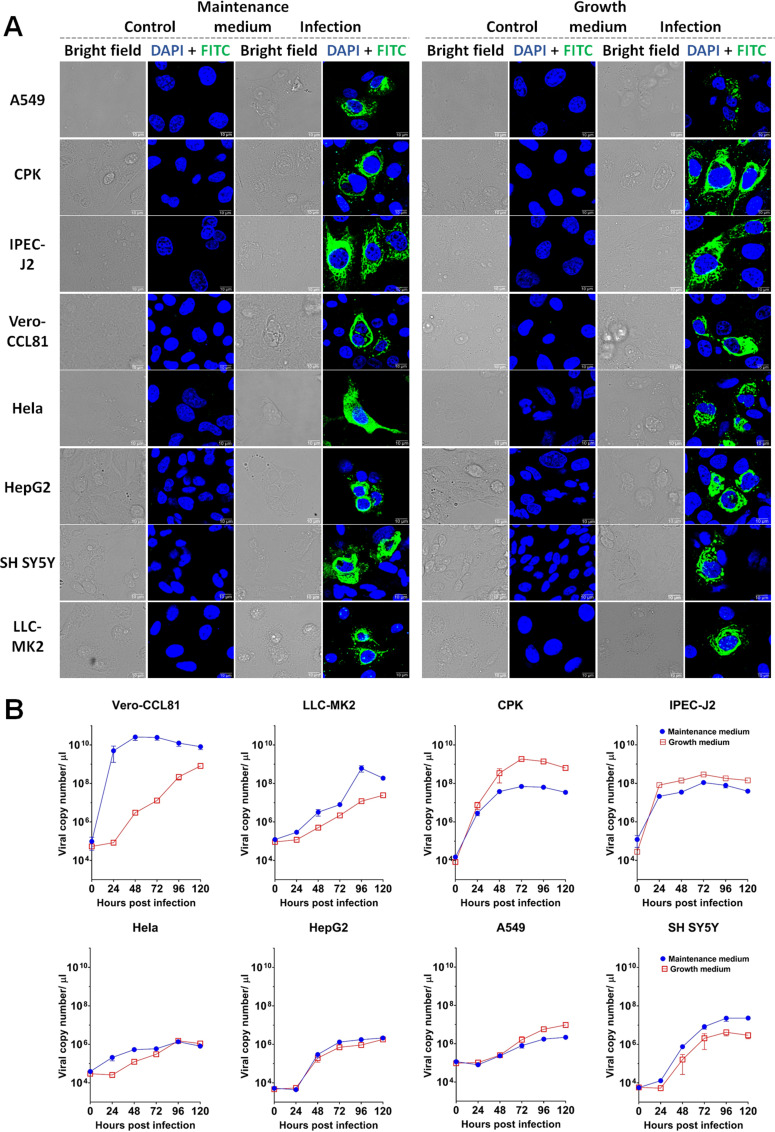
Cell susceptibility examination of BatMRV2/SNU1/Korea/2021. Human-originated cell lines (A549, HepG2, HeLa, and SH-SY5Y) and animal-originated cell lines (Vero-CCL81, LLC-MK2, IPEC-J2, and CPK) were infected with a virus at an MOI of 1 in maintenance media (Dulbecco’s Modified Eagle Medium(DMEM) plus tryptose phosphate broth, yeast extract, and trypsin) or growth media (DMEM supplemented with 10% FBS). (**A**) Immunofluorescence assays were performed after 48 hpi. Mouse anti-BatMRV2/SNU1/Korea/2021 antibodies were applied to detect the viral antigens followed by staining with fluorescein isothiocyanate (FITC)-labeled goat anti-mouse IgG and colored in green. Cell nuclei were stained with 4’,6-diamidino-2-phenylindole (DAPI) and shown in blue. (**B**) Viral growth curves in both media were examined by reverse transcription - quantitative PCR (RT-qPCR) at 0, 24, 48, 72, 96, and 120 hpi. The error bar indicates the standard error.

In other studies, the cell tropism of *Mammalian orthoreovirus* was considered to be strain-dependent (40–42). To examine the range of cells susceptible to our isolate, different cell lines originating from different organs and species were infected at an multiplicity of infection (MOI) of 1. The results indicated that BatMRV2/SNU1/Korea/2021 has a broad cell tropism, including human, non-human primate, and porcine cell lines originating from different organs such as the kidney (Vero, VeroE6, Marc145, LLC-MK2, and CPK), intestine (IPEC-J2), lung (A549), cervix (Hela), and neural cell line from bone marrow (SH SY5Y) (Fig. 3A; Fig. S2). Vero was observed to be highly susceptible to viral propagation, followed by CPK and LLC-MK2 cells. Additionally, virus growth was well supported by not only maintenance media, but normal growth media supplemented with fetal bovine serum (FBS) was also approved for virus replication in these cell lines (Fig. 3B). Notably, our strain replicated better in the maintenance media [basal media supplemented with tryptose phosphate broth (TBP), yeast extract (YE), and trypsin] in most human and non-human primate cell lines, with the exception of A549 cell lines, while in the remaining tested cell lines, viral growth favored the growth media (basal medium supplemented with 10% FBS) (Fig. 3B; Fig. S2). Furthermore, kidney-related cell lines and IPEC-J2, which originated from the intestine of swine, were highly conducive to viral infection, whereas HeLa cells, a cervical cell line, did not adequately support the growth of the virus (Fig. 3B).

### Intranasal but not oral artificial infection causes disease in a mouse model

**Fig 4:**
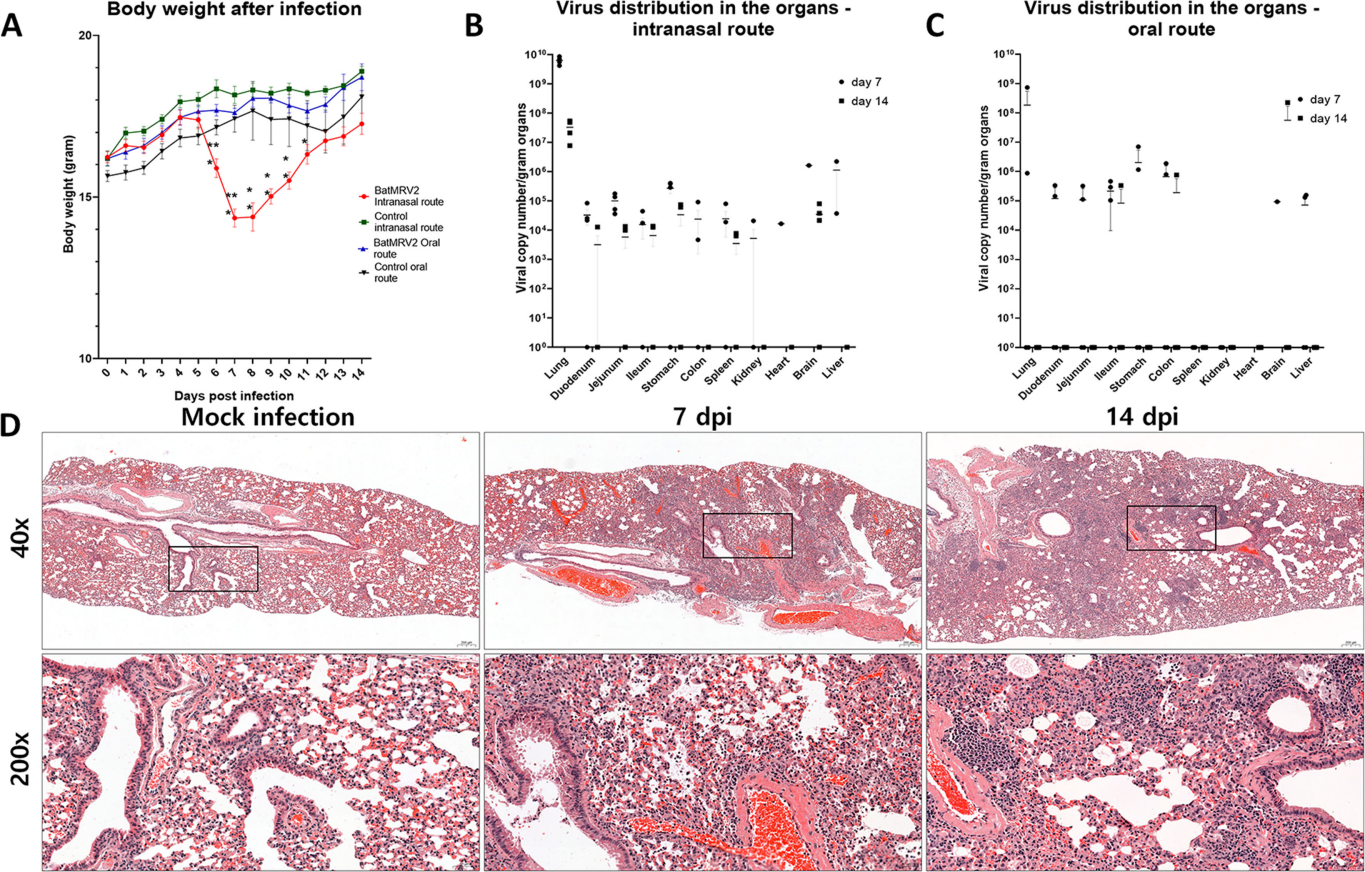
Pathogenicity of BatMRV2/SNU1/Korea/2021 in a mouse model. (**A**) Body weight changes after viral infection. Four-week-old female BALB/c mice were inoculated with 10^6^ TCID_50_ of bat virus via intranasal and oral routes. Body weights were measured daily for a 14-day period. Viral replication was detected in the organs (lung, stomach, duodenum, jejunum, ileum, colon, kidney, liver, spleen, heart, and brain) at day 7 and day 14 in the mouse infected intranasally (**B**) and orally (**C**). (**D**) BatMRV2/SNU1/Korea/2021 caused severe pneumonia in mice infected via the intranasal route. The right lung lobes of mice at day 7 and day 14 were used for pathological examination by hematoxylin–eosin (H-E) staining. Tested tissues showed severe pneumonia with the infiltration of monocyte cells.

Fecal-to-oral and aerosol are the main transmission pathways of mammalian orthoreoviruses (43, 44). To elucidate the pathogenicity of this strain in a mouse model, experimental mice were intranasally or orally inoculated with 10^6^ 50% tissue culture infectious dose (TCID_50_). Control mice were administered through the same route using mock-infected cell supernatants. Mice in all infected groups showed clinical symptoms such as piloerection and huddling behavior from 4 to 9 dpi, whereas all control groups showed normal behavior. However, respiratory distress and weight loss were only observed in the intranasally infected group. Infected mice displayed loss of body weight on days 6 and 7 before gradually recovering to normal weight from days 8 to 11 (Fig. 4A).

To further examine the spread of new isolates throughout the organs in infected mice, the internal organs including the respiratory system, digestive system, and brain were used for RNA extraction and virus detection via the RT-qPCR method. In the intranasal route, the virus grew well in lung samples, achieving approximately 6.4 × 10^9^ copy number per gram organ at 7 dpi, followed by a reduction to 3.3 × 10^7^ copy number per gram of organ at 14 dpi. Virus replication was also detected in all other organs in at least two mice at 7 dpi, but not in the kidneys, heart, or liver, and only in the duodenum of one mouse after 14 dpi (Fig. 4B). In contrast, in the oral route, viral infection in the internal organs was primarily observed at 7 dpi in the examined organs, but not in the spleen, kidney, or heart. At 14 dpi, the virus was only detected in the ileum, colon, and brain. In fact, the viral titer was low and depended on the individual (Fig. 4C).

To examine the gross pathology caused by infection, fixed tissues were sectioned and stained with hematoxylin & eosin stain (H-E). Pneumonia was observed in the lung samples after intranasal inoculation. Multiple infiltrates of inflammatory cells, composed mainly of monocytes, were observed around the bronchioles and blood vessels in the lungs (Fig. 4D). Additionally, the heart sample from one mouse at 7 dpi showed focal cardiac mineralization (Fig. S3). In the oral route group, mild injury to the lungs and stomach was observed at 7 and 14 dpi, respectively (Fig. S3).

### Intranasal infection caused a change in the cytokine and chemokine expression profile in different organs

**Fig 5:**
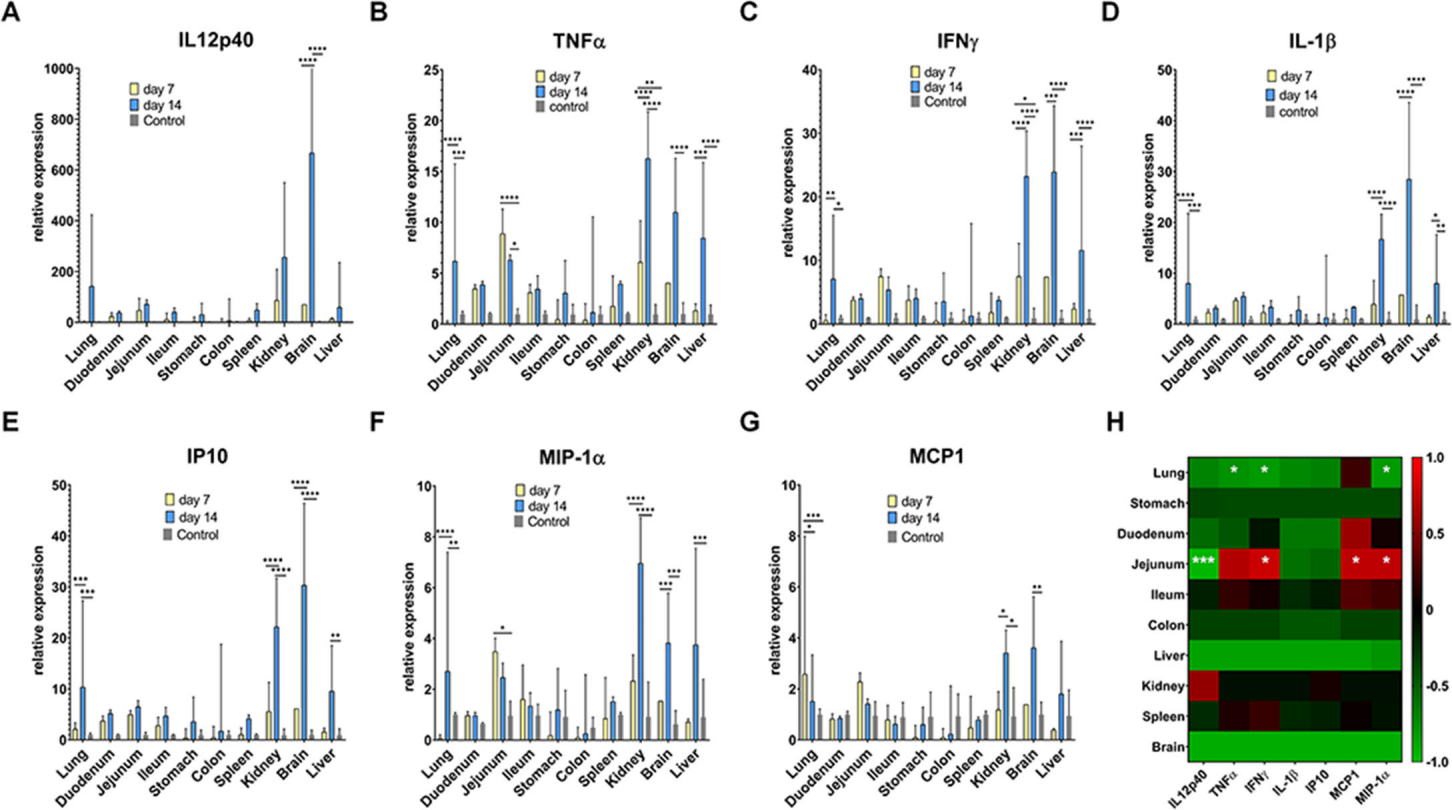
BatMRV2/SNU1/Korea/2021 infection caused a change in the innate immune response. Expression level of cytokines and chemokines (**A**) IL12p40; (**B**) TNFα, (**C**) IFNγ, (**D**) IL-1β, (**E**) IP10, (**F**) MIP-1α, and (**G**) MCP1 in different organs at day 7 and day 14 in infected and control mice examined by RT-qPCR. The error bar indicated the standard error. (**H**) Heatmap analysis indicated the correlation analysis of the expression of cytokines and virus titration in each organ. *P < 0.05; **P < 0.01; ***P < 0.001; ****P < 0.0001.

Due to the clear pathological observation in the intranasal inoculation, the pro-inflammatory cytokine and chemokine responses including interleukin-1β (IL-1β), IL12p40, tumor necrosis factor-alpha (TNFα), interferon-gamma (IFNγ), interferon gamma-induced protein 10 (IP10), monocyte chemoattractant protein 1 (MCP1), and macrophage inflammatory protein 1-alpha (MIP-1α) in each organ of mice in this group were examined at the RNA level. Intriguingly, the results showed a negligible change in the transcription level of almost all examined cytokines in the examined tissues at day 7, except for a significant increase of TNFα and IFNγ expression in the liver (Fig. 5A through G). However, a clear upregulation of these genes was observed in the brain on day 14 (Fig. 5A through G). With the exception of IL12p40, other cytokines were upregulated in the kidney, liver, and lungs (Fig. 5B through G). The transcription levels of cytokines in other digestive tissues and the spleen did not change during the designated period (Fig. 5A through G).

To further investigate the relationship between the cytokine response and virus titer, Spearman’s correlation analysis was conducted for assessing the change between cytokine expression and virus titer in the organs of each individual. A strong inverse correlation between TNFα (*r* = −0.7619), IFNγ (*r* = −0.8095), and MIP-1α (*r* = −0.8095) with the titer of virus was observed in lung samples (*P* < 0.05) (Fig. 5H). A significant inverse relationship (*r* = −0.9581; *P* < 0.001) was observed between IL12p40 mRNA levels and virus replication proficiency, while the increase in IFNγ, MCP1, and MIP-1α mRNA was positively correlated with viral reproduction in the jejunum (*r* = 0.7904, 0.7425, and 0.7425, respectively, *P* < 0.05) (Fig. 5H).

## DISCUSSION

Recently, the diversity of bat viruses has been of interest to many studies because of the high prevalence of disease-related viral families in bats, such as Coronaviridae, Paramyxoviridae*,* and Rhabdoviridae (23, 45, 46). Circulating MRVs in bat populations have been reported in many countries on different continents (30, 32, 37, 47), suggesting pervasive dissemination. Demonstration of serious infections in humans, mice, and swine with bat-borne reoviruses indicates that bats are important intermediate hosts of these viruses (23, 24, 38). Furthermore, genetic analysis of bat reoviruses has revealed that the complex reassortment of segments from different hosts is a common phenomenon (6, 8, 31). Therefore, active surveillance of bat-borne viruses with isolation efforts is crucial to fill the knowledge gap regarding the role of bats in zoonotic spillovers. In this study, we attempted to isolate viruses from different organs taken from carcasses of *M. aurascens*. A bat mammalian orthoreovirus was successfully isolated from the small intestine with a clear CPE, whereas other organs, such as the lung, stomach, liver, and spleen, showed normal morphology. No other viruses were detected using family-specific primers or NGS. Our results are in agreement with those of a previous study by Yan et al., which indicated that bat intestinal samples showed the highest positive rate of MRVs (6).

MRVs are classified into four serotypes based on the sequence of the Sigma 1 encoding gene (S1) (3). Phylogenetic construction using the S1 sequence showed that our strain belonged to serotype 2, which is closely related to other strains circulating in the Japanese population (3, 48). The MRV serotype 2 has also been documented as a cause of acute necrotizing encephalopathy in children in France (12). Therefore, the pathogenicity of this new isolate in humans requires further investigation.

Genomic redistribution is one of the most important evolutionary pathways in segmented viruses (49). The microbat *M. aurascens* has a wide range of natural niches that overlap with human and livestock habitats, thereby increasing the risk of zoonosis and reverse zoonosis. In this study, only the L2 and M3 sequences of the isolated strain were closely related to those from other bat-borne MRV samples, whereas the remaining segments might have originated from variable sources (Table 1; Fig. 2C). Particularly, the S1 and M2 segments, the two major outer viral structural proteins, are highly similar to those originating from humans and swine, respectively (3, 48, 50). The origination of other segments was still unknown, with high homology with those in different sources. In our study, the phylogenetic construction based on each segment indicated a complex relationship between this isolate and other published strains. Specifically, the BatMRV2/SNU1/Korea/2021 segments did not fall within a specific cluster; however, the frequency was exchanged with those of other hosts. This result suggested that numerous reassortment events have appeared during the evolution of batMRV2 in Korea. Moreover, bat, with its specific characteristics, may act as “mixing vessel” of MRVs, resulting in creating new, more virulent disease-causing MRV strains in humans and livestock. These data are consistent with the recent findings of a reassortant bat MRV isolated in China (6). Moreover, recently, a bat MRV1 strain containing heterogeneous segments from bat, human, rodent, and swine MRVs was isolated in Korea (37). Our study, in combination with the aforementioned studies, provides evidence of complex segment mixtures from different sources of MRVs in bats.

The phylogenetic network is a powerful tool in evolutionary studies that considers recombinant and horizontal gene transfer among populations (51). Recently, this approach has been widely applied in the evolution of, for example, SARS-CoV2 and influenza virus (52–56). In the influenza virus, a segmented negative-strand virus, there is a high rate of reassortment during the evolution of all circulating influenza A virus serotypes H1N1, pH1N1, H2N2, H3N2, and influenza B virus (55). In this study, we applied this approach to identify the reassortment events that occurred during the evolution of this strain. As expected, at least two gene flow events were related to our strain, and 12 other events occurred (Fig. 2B). However, because of the limitations in the whole-genome sequence of MRVs available in GenBank, we cannot rule out the genetic drift of unidentified MRV circulation in bats or other hosts. This result provides the initial evidence of gene flow events during the evolution of MRVs.

Bat MRVs have been shown to exhibit a broad range of cell susceptibility (6, 33, 47). In this study, the collected strain was examined for potential infectivity in different cell lines from different organs, such as the kidney, lung, intestine, cervix, and bone, from a variety of hosts, including humans, non-human primates, and pigs. This result, in agreement with those of previous studies, showed that BatMRV2/SNU1/Korea/2021 can infect many different cell lines, although they have different replication kinetics (Fig. 3; Fig. S2). Several cell lines, such as MDCK, Vero, Marc145, and PK15, are normally used for isolation of MRVs using media supplemented with either FBS or trypsin (6, 37, 47, 57). Therefore, we compared the growth kinetics of our strain in each cell line in different media. Interestingly, trypsin-supplemented media promoted viral replication in most human and non-human primate cell lines, whereas growth media were suitable for viral growth in human and porcine lung cell lines (Fig. 3B). In previous studies, trypsin was shown to contribute to the infectivity of viruses in different steps, such as entry, cell-to-cell transmission, or event virus production of coronaviruses and herpesvirus (58–60). However, the effects of trypsin on the infectivity of MRVs have not been investigated. Additionally, the influence of other components such as TBP or YT on MRV reproduction should not be excluded. Nevertheless, to the best of our knowledge, this is the first report comparing the propagation of MRVs using different media.

MRVs have been previously reported to cause gastrointestinal and respiratory problems in experimental animals (7, 33, 47). Natural infections caused by MRVs include aerosol and/or oral transmission. Therefore, we examined two routes of infection: intranasal and intragastric, at a dose of 10^6^ TCID_50_. Our results showed that the BatMRV2/SNU1/Korea/2021 strain was able to infect and cause diseases in mice via the intranasal route, inducing weight loss, respiratory distress (Fig. 4A), and extensive lung infection (Fig. 4D). These results are consistent with those of previous studies, indicating that MRVs can cause serious pneumonia in mice (33, 61). However, in both infection routes, the virus was detected in different organs, with the highest titer in the lungs. Interestingly, even when isolated from intestinal samples, the gastrointestinal tract was not well supported by viral infection, even when inoculated via the oral route (Fig. 4C). Furthermore, viruses were detected in the brains of the experimental mice (Fig. 4B and C). Considering that BatMRV2/SNU1/Korea/2021 can infect SH-SY5Y cells, we hypothesized that this virus has a neurotropic capacity.

The cytokine and chemokine responses are the most important immune reactions against viral infections. Different MRV strains induce different kinds of innate immune responses (33). These cytokine responses may have double-edged effects. On the one hand, they can reduce viral infection; on the other hand, overproduction of cytokines/chemokines may be correlated with organ injury (62, 63). In this study, BatMRV2/SNU1/Korea/2021 caused a delayed response, with a clear increase in cytokine and chemokine expression at 14 dpi. Although the clear occurrence of pneumonia was observed in the lung samples at 7 dpi, only MCP1 showed a significant increase at this time point, whereas other pro-inflammatory cytokine and chemokine transcription levels were observed to have increased at 14 dpi. Furthermore, although a notable increase in cytokine and chemokine responses was observed in the brain, kidneys, and liver, no histopathological changes were observed in these organs during the experimental period. The expression levels of cytokines observed in this study contradicted the findings of a previous report that demonstrated that the cytokine response increased at 7 dpi in lung samples (33). In addition, our study revealed that TNFα, IFNγ, and MIP-1α were beneficial for viral clearance in lung samples. TNFα and IFNγ were demonstrated as having an anti-viral effect against different types of viruses (64–66).

In this study, we isolated and characterized the genetics and pathogenicity of a new *Mammalian orthoreovirus* type 2 strain originating from a bat. Whole-genome sequence comparison and phylogenetic analysis indicated that this strain may have resulted from the mixing of genetic material from humans, pigs, and bats. *In vitro* and *in vivo* experiments indicated that this strain could infect and replicate in different cell lines and cause diseases in mice, suggesting its spillover capability. Additionally, the immune response of mice to this virus was investigated. Our study contributes to further understanding of the evolution of bat MRVs and the risk to public and animal health. Future studies should focus on the diversity of MRVs in wildlife and the risk of spillover transmission.

## MATERIALS AND METHODS

### Virus isolation

In a previous study, we collected six carcasses of different bat species (67). For virus isolation, organ samples (brain, lung, heart, liver, stomach, intestine, kidney, and spleen) were homogenized in Dulbecco’s Modified Eagle Medium (DMEM), filtered, and inoculated in Vero-CCL81 cells. After 2-h absorption, the fresh maintenance medium was added (DMEM supplemented with 0.3% TBP, 0.02% YE, and 4 µg/ml trypsin). The inoculated cells were observed daily for CPE for a 5-day period. If no CPE was observed, infected cells were frozen and thawed three times and clarified by centrifuging at 3,720 × *g*/4°C for 20 minutes. The samples were serially passaged up to five times in the same cell type for CPE monitoring. Family-specific primers against Coronaviridae*,* Paramyxoviridae*,* and Adenoviridae were used to screen for virus-infected tissues (68–70). To further detect the unknown virus, particle-associated nucleic acid PCR (71) was used, with some modifications, as described previously (72). Sequences obtained by sequencing were compared with sequences available in GenBank to identify the viruses.

### Virus characterization

For transmission electron microscopy, the viral supernatant was centrifuged at 12,000 rpm/4°C for 30 minutes to remove any remaining cell fragments. The virus was further concentrated in an Amicon 50 kDa (Merck Millipore, Germany), followed by fixation with 0.1% formaldehyde for 24 h. Samples were negatively stained and examined using a JEM-F200 (Japan Electron Optics Laboratory, Tokyo, Japan) at 100,000× magnification.

To determine the temperature sensitivity, the virus was incubated at 4°C (control), 37°C, 56°C, 80°C, and 90°C for 1 h. Temperature-treated and control viral solutions were serially diluted 10 times and titrated in Vero-CCL-81 cells. Viral infection was determined by observing the CPE using a light microscope. TCID_50_ was calculated using the Spearman–Karber method.

For the double-stranded RNA migration test, the viral solution was treated with DNase and RNase for 1 h, and viral RNA was extracted from the viral particles using the QIAamp Viral RNA Mini Kit (QIAGEN, USA). Genomic RNA was separated by exposure to a constant current of 1 mA on double layers of 8% and 12% SDS-PAGE for 22 h. RNA was stained using SafeView (Intron Biotech, Seoul, Korea) and visualized under UV light.

### Cell tropism test

African green monkey kidney Vero E6, monkey kidney cells LLC-MK2, African green monkey kidney cell Marc145, porcine kidney cell CPK, intestine porcine epithelial cell IPEC-J2, human alveolar basal epithelial cell A549, human cervix cells Hela, and human neuroblastoma cell SH-SY5Y were cultured in DMEM plus 10% FBS (growth media); the human liver cell line HepG2 was grown in Roswell Park Memorial Institute (RPMI) 1640 medium plus 10% FBS (growth media). Maintenance medium was applie for these cells using the same basal media supplemented with 0.3% TBP, 0.02% YE, and 1 µg/ml trypsin, except the SH-SY5Y were used 0.1 µg/ml trypsin. All cells were maintained at 37°C and 5% CO_2_.

For the cell tropism test, the virus was allowed to absorb for 1 h at 37°C, and then the cells were washed twice with PBS 1× and cultured in the growth medium or maintenance medium for 24 h. Cells were fixed with 4% formaldehyde, permeabilized with 0.1% Triton-X100 in PBS, and blocked with 5% skim milk. Viral amplification was performed using mouse anti-BatMRV2/SNU1/Korea/2021 polyclonal antibodies, followed by staining with fluorescein isothiocyanate (FITC)-labeled goat anti-mouse antibodies (Abcam, USA) and 4’,6-diamidino-2-phenylindole (DAPI) (Invitrogen, USA). The images were acquired by using a TCS8 confocal microscope (Leica Biosciences).

The growth curve of the virus was determined using RT-qPCR, as described below. Briefly, 200 µl of viral solutions at 0, 24, 48, 72, 96, and 120 dpi was collected and used for RNA extraction. RT-qPCR was performed using a Luna Universal Probe One-Step RT-qPCR Kit (NEB, USA) to determine the viral load.

### Whole-genome sequencing and phylogenetic analysis

The viral solution was treated with DNase and RNase to remove host genetic material, followed by RNA extraction. Paired-end whole RNA sequencing (101 bp) was performed by Macrogen using the Illumina platform. After low-quality read filtration using Trimmomatic v0.39 (73), the viral genome was *de novo* assembled using SPAdes v3.15 (74). Viral genome contigs were BLASTed and compared with the MRV sequences available in GenBank. The bat MRV genome was further polished by mapping the raw reads to the assembled genome using BWA v0.7 (75) and Samtools v1.10 (76).

The ed segments were compared with those of other complete sequences of mammalian orthoreoviruses using Mafft v7.471 (77). Phylogenetic construction was performed using Iqtree with the best-fit substitution model automatically selected by option “-m MFP” (78). Branch support was calculated using the ultrafast bootstrap approximation (79). The constructed tree was displayed using FigTree v1.4.4 (https://github.com/rambaut/figtree/ accessed on 9 September 2022). A phylogenetic network was constructed using RF-Net2 (80), with a phylogenetic tree of 10 segments as the input. Phylogenetic networks between our strain and other strains were generated by Icytree (https://icytree.org/) (81). The reassortment event was examined using RPD v5.34 (82) with RPD, GENECONV, Chimera, MaxChi, and BootScan algorithms and further visualized using SimPlot v.3.5.1 (83)

### Animal infection experiments

Four-week-old female BALB/c mice were inoculated intranasally or intragastrically with 10^6^ TCID_50_ or lysed mock-infected cells as controls. Clinical symptoms and body weight were measured daily for 14 days. Experimental mice and control mice were euthanized with carbon dioxide on 7 and 14 dpi. Tissues, including the lung, heart, liver, stomach, duodenum, jejunum, ileum, colon, kidney, spleen, and heart, were collected and stored in 10% buffered paraformaldehyde for H-E staining or RNAlater (Thermo Scientific, USA) for RNA extraction.

Total RNA was extracted from the tissues using the QIAgen RNA Mini Kit (QIAGEN, USA). Virus titers were determined by RT-qPCR, as described above. cDNA was synthesized using the Tetra cDNA Synthesis Kit (Meridian Bioscience, Korea) with polyT primers. The expression of cytokines (IL-1β, IL12p40, TNFα, and IFNγ) and chemokines (IP1, MCP1, and MIP-1α) was determined using the Luna Universal qPCR Master Mix (NEB, USA). Beta-actin RNA was used as an internal control to normalize the input cDNA.

### Statistical analyses

Statistical analyses were performed using GraphPad Prism v.8.0.2. Significant differences between groups were measured using two-way ANOVA. The correlation between cytokines/chemokines and virus titers was determined using Spearman’s correlation.

## Data Availability

The sequences of BatMRV2/SNU1/Korea/2021 described in this study have been deposited in GenBank under accession numbers OQ789607 to OQ789616.

## SUPPLEMENTAL MATERIAL

Fig. S1 spectrum.01762-23-s0001.doc Phylogenetic construction of segments (A) L1, (B) L2, (C) L3, (D) M1, (E) M2, (F) M3, (G) S1, (H) S2, (I) S3, and (J) S4. The color and width of the node shape indicate the bootstrap values. BatMRV2/SNU1/Korea/2021 are shown in red.

Fig. S2 spectrum.01762-23-s0002.doc Cell susceptibility examination of BatMRV2/SNU1/Korea/2021. Marc145 and Vero-E6 cells were infected with the virus at MOI=1 in maintenance medium (DMEM plus trypto-phosphate broth, yeast extract, and trypsin) or growth media (DMEM supplemented with 10% FBS). (A) Immunofluorescence assay was performed at 48 hpi. Mouse anti-BatMRV2/SNU1/Korea/2021 antibodies were used to detect the viral antigens, followed by staining with FITC-labelled goat anti-mouse IgG (green). The cell nuclei were stained with DAPI and are shown in blue. (B) Viral growth curves in both media were examined using RT-qPCR at 0, 24, 48, 72, 96, and 120 hpi. Error bars indicate standard error.

Fig. S3 spectrum.01762-23-s0003.doc (A) Pathology of the heart of a mouse intranasally infected with BatMRV2/SNU1/Korea/2021. The result showed Focal cardiac mineralization was observed. (B) Mononuclear cell infiltration and focal and minimal to mild infiltration were observed in the stomachs of mice infected with BatMRV2/SNU1/Korea/2021 via oral route. (C) Pathology of lung samples from mouse infected with BatMRV2/SNU1/Korea/2021 via oral route. Mononuclear perivascular and peribronchiolar inflammation were also observed. Mild-to-moderate multifocal injuries are also observed. The regions related to the histopathology of the heart and stomach were marked.

ASM does not own the copyrights to Supplemental Material that may be linked to, or accessed through, an article. The authors have granted ASM a non-exclusive, world-wide license to publish the Supplemental Material files. Please contact the corresponding author directly for reuse.
