# Supplementary material for: Genetic characterization and pathogenicity in a mouse model of newly isolated bat-originated mammalian orthoreovirus in South Korea
Source: Microbiol Spectr. 2024 Jan 30;12(3):e01762-23. doi: 10.1128/spectrum.01762-23 (PMC10913406; doi:10.1128/spectrum.01762-23)
Supplement: Fig. S1 — Phylogenetic construction of segments (A) L1, (B) L2, (C) L3, (D) M1, (E) M2, (F) M3, (G) S1, (H) S2, (I) S3, and (J) S4. The color and width of the node shape indicate the bootstrap values. BatMRV2/SNU1/Korea/2021 are shown in red. [file spectrum.01762-23-s0001.doc]

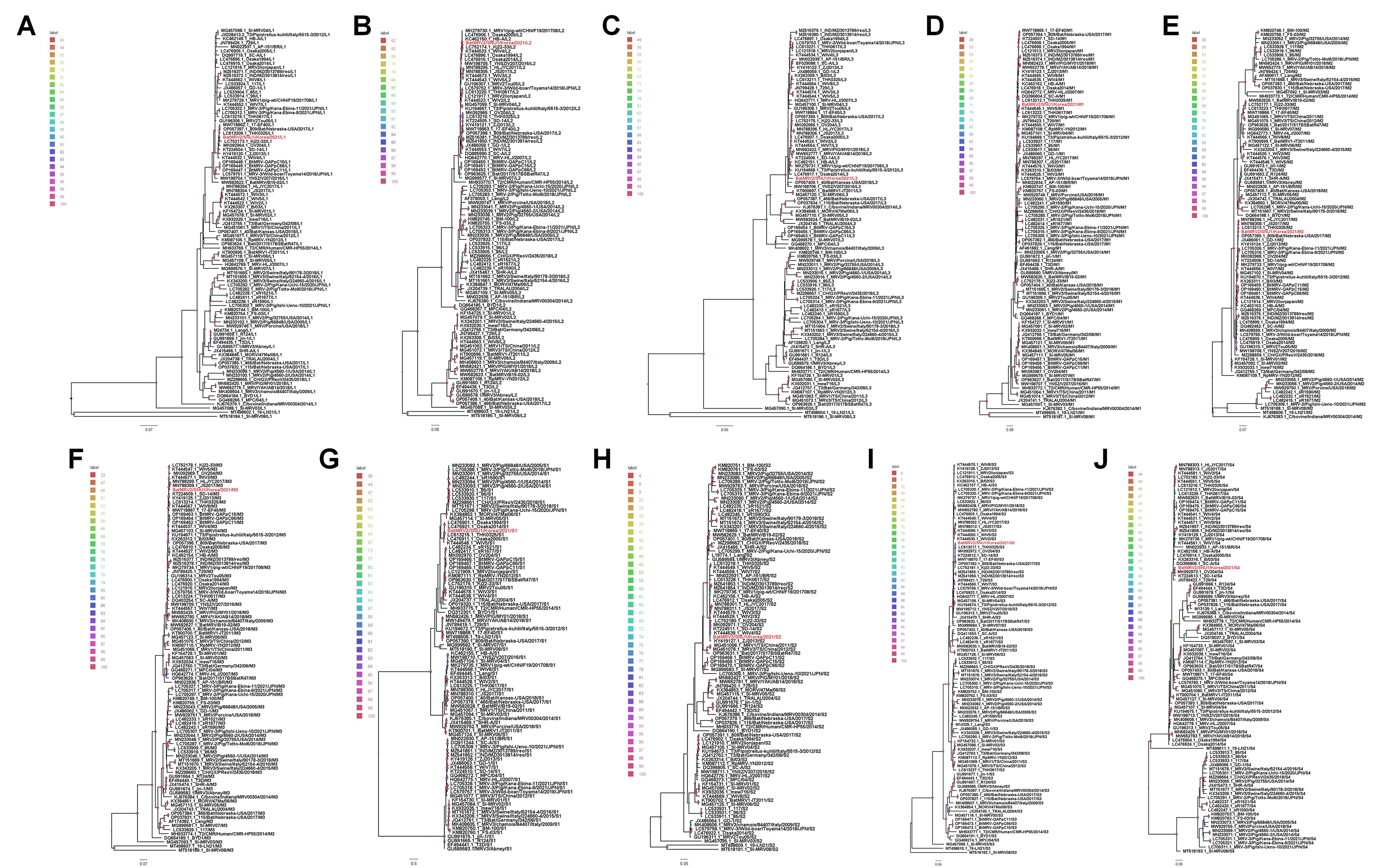


Fig. S1. Phylogenetic construction of segments (A) L1, (B) L2, (C) L3, (D) M1, (E) M2, (F) M3, (G) S1, (H) S2, (I) S3, and (J) S4. The color and width of the node shape indicate the bootstrap values. BatMRV2/SNU1/Korea/2021 are shown in red.
