# Supplementary material for: Genetic characterization and pathogenicity in a mouse model of newly isolated bat-originated mammalian orthoreovirus in South Korea
Source: Microbiol Spectr. 2024 Jan 30;12(3):e01762-23. doi: 10.1128/spectrum.01762-23 (PMC10913406; doi:10.1128/spectrum.01762-23)
Supplement: Fig. S2 — Cell susceptibility examination of BatMRV2/SNU1/Korea/2021. Marc145 and Vero-E6 cells were infected with the virus at MOI=1 in maintenance medium (DMEM plus trypto-phosphate broth, yeast extract, and trypsin) or growth media (DMEM supplemented with 10% FBS). (A) Immunofluorescence assay was performed at 48 hpi. Mouse anti-BatMRV2/SNU1/Korea/2021 antibodies were used to detect the viral antigens, followed by staining with FITC-labelled goat anti-mouse IgG (green). The cell nuclei were stained with DAPI and are shown in blue. (B) Viral growth curves in both media were examined using RT-qPCR at 0, 24, 48, 72, 96, and 120 hpi. Error bars indicate standard error. [file spectrum.01762-23-s0002.doc]

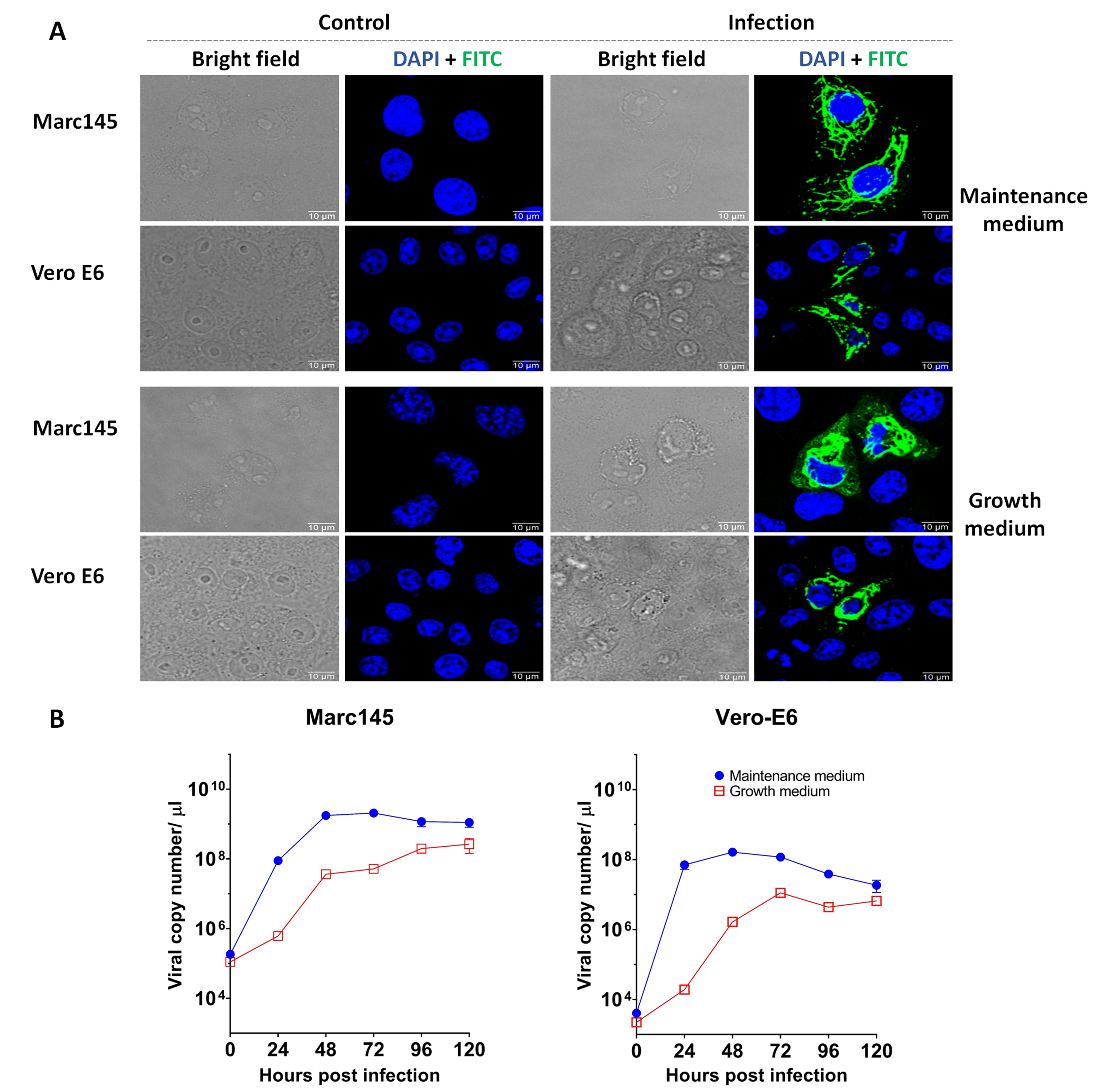


Fig. S2 Cell susceptibility examination of BatMRV2/SNU1/Korea/2021. Marc145 and Vero-E6 cells were infected with the virus at MOI=1 in maintenance medium (DMEM plus trypto-phosphate broth, yeast extract, and trypsin) or growth media (DMEM supplemented with 10% FBS). (A) Immunofluorescence assay was performed at 48 hpi. Mouse anti BatMRV2/SNU1/Korea/2021 antibodies were used to detect the viral antigens, followed by staining with FITC-labelled goat anti-mouse IgG (green). The cell nuclei were stained with DAPI and are shown in blue. (B) Viral growth curves in both media were examined using RT-qPCR at 0, 24, 48, 72, 96, and 120 hpi. Error bars indicate standard error.
