# Supplementary material for: Genetic characterization and pathogenicity in a mouse model of newly isolated bat-originated mammalian orthoreovirus in South Korea
Source: Microbiol Spectr. 2024 Jan 30;12(3):e01762-23. doi: 10.1128/spectrum.01762-23 (PMC10913406; doi:10.1128/spectrum.01762-23)
Supplement: Fig. S3 — (A) Pathology of the heart of a mouse intranasally infected with BatMRV2/SNU1/Korea/2021. The result showed Focal cardiac mineralization was observed. (B) Mononuclear cell infiltration and focal and minimal to mild infiltration were observed in the stomachs of mice infected with BatMRV2/SNU1/Korea/2021 via oral route. (C) Pathology of lung samples from mouse infected with BatMRV2/SNU1/Korea/2021 via oral route. Mononuclear perivascular and peribronchiolar inflammation were also observed. Mild-to-moderate multifocal injuries are also observed. The regions related to the histopathology of the heart and stomach were marked. [file spectrum.01762-23-s0003.doc]

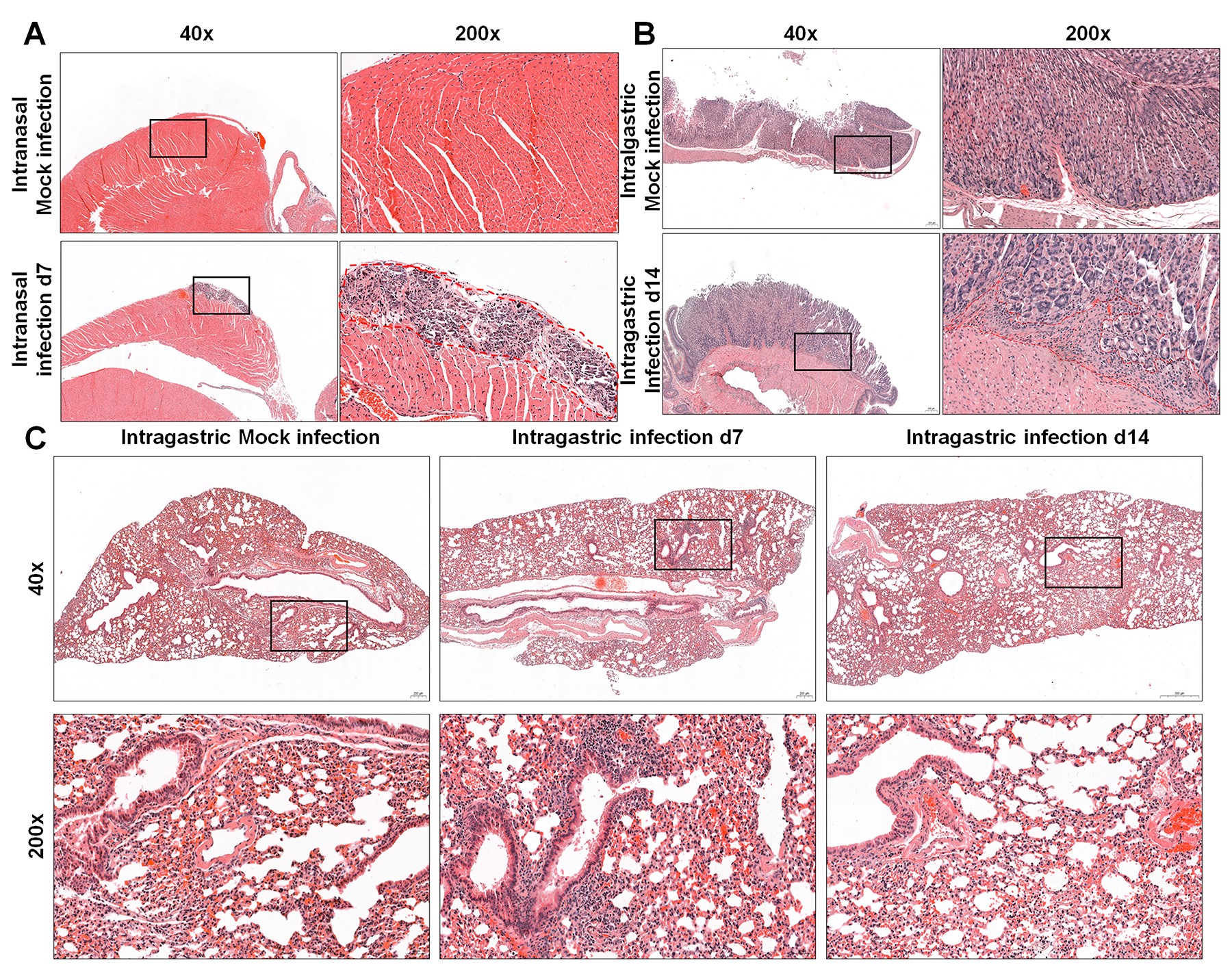


Fig. S3 (A) Pathology of the heart of a mouse intranasally infected with BatMRV2/SNU1/Korea/2021. The result showed Focal cardiac mineralization was observed. (B) Mononuclear cell infiltration and focal and minimal to mild infiltration were observed in the stomachs of mice infected with BatMRV2/SNU1/Korea/2021 via oral route. (C) Pathology of lung samples from mouse infected with BatMRV2/SNU1/Korea/2021 via oral route. Mononuclear perivascular and peribronchiolar inflammation were also observed. Mild-to-moderate multifocal injuries are also observed. The regions related to the histopathology of the heart and stomach were marked.
